# AMA- and RWE- Based Adaptive Kalman Filter for Denoising Fiber Optic Gyroscope Drift Signal

**DOI:** 10.3390/s151026940

**Published:** 2015-10-23

**Authors:** Gongliu Yang, Yuanyuan Liu, Ming Li, Shunguang Song

**Affiliations:** 1 School of Instrument Science and Opto-Electronics Engineering, Beihang University, Beijing 100191, China; E-Mails: bhu17-yang@139.com (G.Y.); liliyalm@buaa.edu.cn (M.L.); 2 Inertial Technology Key Laboratory of National Defense Science and Technology, Beihang University, Beijing 100191, China; 3 Beijing Institute of Spacecraft System Engineering, Beijing 100094, China; E-Mail: songshunguang@126.com

**Keywords:** Adaptive Moving Average (AMA), Random Weighting Estimation (RWE), Fiber Optic Gyroscope (FOG), Kalman Filter (KF)

## Abstract

An improved double-factor adaptive Kalman filter called AMA-RWE-DFAKF is proposed to denoise fiber optic gyroscope (FOG) drift signal in both static and dynamic conditions. The first factor is Kalman gain updated by random weighting estimation (RWE) of the covariance matrix of innovation sequence at any time to ensure the lowest noise level of output, but the inertia of KF response increases in dynamic condition. To decrease the inertia, the second factor is the covariance matrix of predicted state vector adjusted by RWE only when discontinuities are detected by adaptive moving average (AMA).The AMA-RWE-DFAKF is applied for denoising FOG static and dynamic signals, its performance is compared with conventional KF (CKF), RWE-based adaptive KF with gain correction (RWE-AKFG), AMA- and RWE- based dual mode adaptive KF (AMA-RWE-DMAKF). Results of Allan variance on static signal and root mean square error (RMSE) on dynamic signal show that this proposed algorithm outperforms all the considered methods in denoising FOG signal.

## 1. Introduction

As a kind of inertial sensor based on optical Sagnac effect, fiber optic gyroscope (FOG) has been widely used in inertial navigation system (INS) because of its significant advantages such as small size, low cost, no moving parts, long lifespan, and large dynamic range [1,2]. However, the accuracy of the FOG sensor is limited by drift errors due to internal device operations and external environment disturbances [3]. The FOG drift signal has two types of errors namely, deterministic errors and stochastic errors. Deterministic errors are bias, scale factor, and misalignment, which are relatively easier to be compensated by suitable calibration methods in laboratory environment. Stochastic errors, which are induced from the environmental temperature changes, electronic noises, and other electronic equipment interfaced with it [4,5], are difficult to be directly eliminated. As an alternative, stochastic models and denoising methods are two main techniques to restrain the FOG random errors reported in the literature.

Signal processing methods like low pass filter [6], wavelet transforms [7,8], and empirical mode decomposition (EMD) [9,10,11] are used to remove random errors for improving the performance of FOG measurement. These methods are successfully applied for denoising FOG static signal but fail to denoise FOG signal in highly dynamic conditions due to delay or complexity. Recently, time series analysis as a powerful tool is used to model the FOG random drift signal. Autoregressive (AR), moving average (MA), and autoregressive moving average (ARMA) have been developed in modeling stochastic models for FOG random errors in [12,13,14]. Combining these stochastic models, a conventional Kalman filter (CKF) is usually employed to remove the FOG random drift [13,14,15,16], where the process and measurement noises are pre-calculated by sampling lots of drift data. However, fixed noise variances are unsuitable in real applications which may lead to divergent problems. To improve the practicability and to avoid divergent effects, adaptive KF (AKF) methods have been investigated which are based on innovation-based adaptive estimation (IAE) or residual-based adaptive estimation (RAE) [17,18,19]. An AKF with double transitive factors is developed in [17,18], where the covariance matrix of predicted state vector is modified by an adaptive factor in stage one and the covariance matrix of measurement noise is modified by another adaptive factor in stage two. However, this method requires that innovation or residual vectors at each time point be in the identical type, spatial dimension and distribution, which is difficult to satisfy in a highly dynamic environment.

Random weighting estimation (RWE) is an advanced computational method in statistics. It does not require the prior knowledge of the distribution of position parameters, and the obtained estimation is unbiased. RWE has been established for estimation of the covariance matrix of observation vector and predicted state vector in [20,21,22]. In [23], Kalman gain based on RWE is updated using the covariance matrix of innovation sequence. This method called RWE-AKFG is applied to denoise the FOG signal under static and dynamic environments. However, the inertia of its response increases in denoising the FOG dynamic signal, which is not tolerable for real time dynamic applications. To solve the contradiction between the noise level of the output signal and the inertia of the KF response, adaptive moving average (AMA) is used to detect the discontinuities in signal [24]. AMA based dual mode KF (AMADMKF) in [25,26] is proposed to filter the FOG static and dynamic signals, where DMKF means the proper KF gain parameter or the ratio *Q/R* is switched at different conditions. However, it is difficult to predefine the proper ratio *Q/R* to adapt the FOG rotation rate changes [27]. Based on the above research, AMA-DWT-DMKF is used to denoise the FOG static signal, disturbance signal, and the change rate signal successfully in [28], but it is much more complex and has a higher computation cost. Considering the nonlinearity in system and measurement models, Narasimhappa *et al.* in [29,30,31] provide various adaptive filters based on unscented Kalman filter such as adaptive unscented Kalman filter (AUKF), adaptive square root unscented Kalman filter (ASRUKF) and adaptive sampling strong tracking scaled unscented Kalman filter (ASST-SUKF) for denoising the FOG signal. The performance of these algorithms is verified in static and dynamic conditions, whereas UKF is more complex than KF. Hence, the purpose of this paper is to develop an AMA- and RWE-based double-factor adaptive KF algorithm, named AMA-RWE-DFAKF, which can denoise the FOG static and dynamic signal. The first adaptive factor is Kalman gain, which is updated by using RWE of the covariance matrix of innovation sequence in any condition. The second adaptive factor is the covariance matrix of predicted state vector, which is revised based on RWE only when the discontinuities are detected by AMA. In fact, it has the same adaptive filter mechanism as [17], except that different estimations are used. Experimental results show that the proposed algorithm can satisfy the lowest noise level and the lowest inertia in denoising FOG static and dynamic signals.

The outline of this paper is as follows: Section 1 is the introduction; the concept of adaptive moving average is reviewed briefly in Section 2; Section 3 gives a description about the principle of random weighting estimation; the proposed AMA-RWE-DFAKF is provided in Section 4; experimental results and discussions about the proposed method applied in FOG static and dynamic signals are presented in Section 5, and Section 6 is the conclusion.

## 2. Concept of Adaptive Moving Average

**Figure 1:**
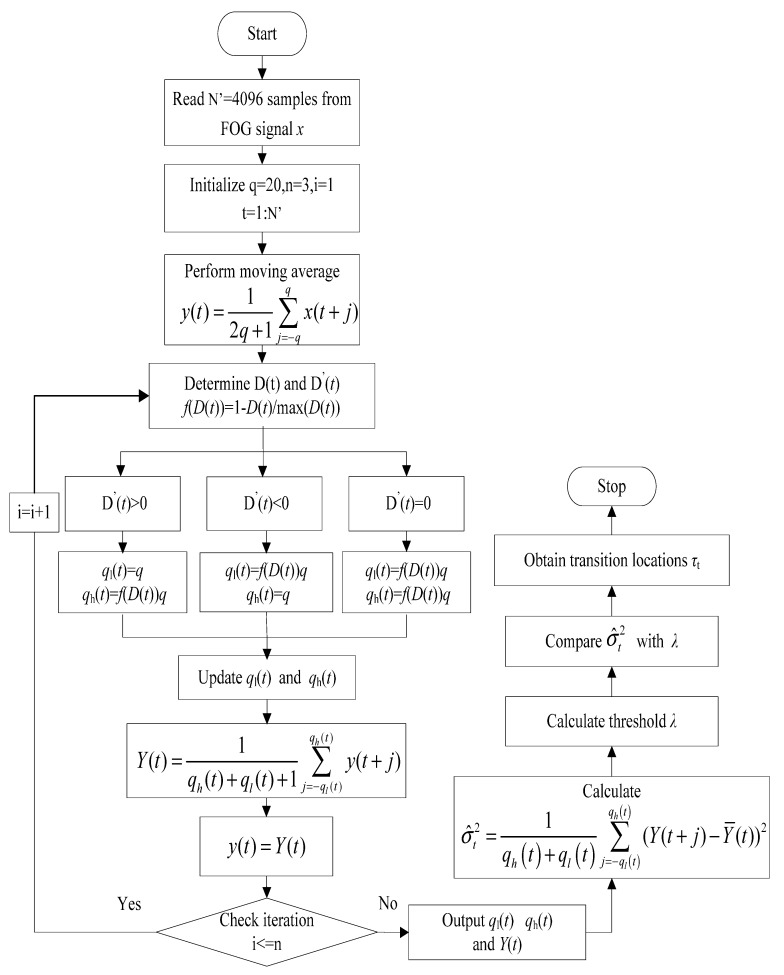
Procedure of the AMA algorithm.

The adaptive moving average (AMA) can be used to detect discontinuities in the signal by comparing the sample variance with a threshold value, where the length of moving average is adaptive to follow the rate of change in signal [24,25,26,28]. A *q*-point moving average filter can be expressed as

(1) $$y(t)=\frac{1}{2q+1}\sum_{j=-q}^{q}x(t+j)\;\;\;\;\;q+1\leq t\leq N-q$$

where *x*(*t*) denotes the input data, 2*q* + 1 is the moving average window size, *N* is the number of samples as one frame of the input data, and *y*(*t*) is the filtered data. The noise can be further reduced by applying an iterative adaptive moving average filter shown in Figure 1. The algorithmic steps of this filter are explained as

Step 1: Calculate the absolute value of the differenced *y*(t)

(2) D(t)=|y(t+q)−y(t−q)|

Step 2: Calculate the rate of change of *D*(t)

(3) $$D^{\prime }(t)=D(t+1)-D(t)$$

Step 3: Calculate the adaptive filtered data *Y*(t)

(4) $$Y(t)=\frac{1}{q_{h}(t)+q_{l}(t)+1}\sum_{j=-q_{l}(t)}^{q_{h}(t)}y(t+j)\begin{array}{cc} & q+q_{l}+1\leq t\leq N-q \end{array}-q_{h}$$

where

(5) $$q_{h}(t)=\{\begin{array}{c} q\\ f(D(t))q \end{array}\begin{array}{cc} & \begin{array}{c} if\begin{array}{c} D^{\prime }(t)<0 \end{array}\\ if\begin{array}{c} D^{\prime }(t)\geq 0 \end{array} \end{array} \end{array}$$

(6) $$q_{l}(t)=\{\begin{array}{c} q\\ f(D(t))q \end{array}\begin{array}{cc} & \begin{array}{c} if\begin{array}{c} D^{\prime }(t)>0 \end{array}\\ if\begin{array}{c} D^{\prime }(t)\leq 0 \end{array} \end{array} \end{array}$$

(7) $$f(D(t))=1-\frac{D(t)}{\max\left(D(t)\right)}$$

Step 4: Repeat the iteration on the filtered data *Y*(*t*) from step 1 to 3 until the maximum iterations is met.

The final filtered data *Y*(*t*) is obtained by *n* iteration in our work, *i.e.*, *n* = 3. And the transition locations are detected by comparing the sample variance of *Y*(*t*) with a threshold *λ*. The sample variance is calculated in the window size as follows

(8) $${\hat{\sigma }}_{t}^{2}=\frac{1}{q_{h}\left(t\right)+q_{l}\left(t\right)}\sum_{j=-q_{l}\left(t\right)}^{q_{h}\left(t\right)}{\left(Y(t+j)-\bar{Y}(t)\right)}^{2}$$

The threshold *λ* is 95% upper tail of exponential distribution, where the expected value of this distribution is the mean value of the above calculated sample variances in the current frame. The transition location *τ*_t_ is defined as

(9) $$\tau_{t}=(t|\begin{array}{c} {\overset{⌢}{\sigma }}_{t}^{2} \end{array}>\lambda ,t=3q+1,3q+2,\cdots N-3q-1)$$

## 3. Principle of Random Weighting Estimation

Suppose that *X*_1_, *X*_2_, … , *X*_n_ are the independent and identically distributed random variables with common distribution function *F*(*x*). Let *x*_1_, *x*_2_, … , *x*_n_ be the sample realizations. The corresponding empirical distribution function *F*_n_(*x*) can be expressed as

(10) $$F_{n}(x)=\frac{1}{n}\sum_{i=1}^{n}I_{\left(X_{i}\leq x\right)}$$

The random weighting estimation [21,22,23] of *F*_n_(*x*) is defined as

(11) $$F_{n}^{*}(x)=\sum_{i=1}^{n}V_{i}I_{\left(X_{i}\leq x\right)}$$

where *I*_(*X*i ≤ **x**)_ is the indicator function represented as

(12) $$I_{\left(X_{i}\leq x\right)}=\{\begin{array}{c} 1\begin{array}{cc} & X_{i}\leq x \end{array}\\ 0\begin{array}{cc} & X_{i}>x \end{array} \end{array}$$

and random vector [*V*_1_, *V*_2_, … , *V*_n_] subjects to Dirichlet distribution *D*(1, 1, … , 1), that is, $\sum_{i=1}^{n}V_{i}=1$. A joint density function of the random vector is defined as

*f*(*V*_1_, *V*_2_, … , *V*_n_) = (n-1)! (13)

where [*V*_1_, *V*_2_, … , *V*_n_] ∈ *D*_n_, and *D*_n-1_={[*V*_1_, *V*_2_, … , *V*_n-1_]: *V*_i_ ≥ 0 (*I* = 1, 2, … ,*n*−1), $\sum_{i=1}^{n-1}V_{i}\leq 1$}.

Calculate the mean of *X*_i_ as follows

(14) $${\hat{E}}_{X}=\frac{1}{n}\sum_{i=1}^{n}X_{i}$$

Then, the random weighting estimation of ${\hat{E}}_{X}$ is

(15) $${\hat{E}}_{X}^{*}=\sum_{i=1}^{n}v_{i}X_{i}$$

Calculate the variance of *X*_i_ as follows

(16) $${\hat{\sum }}_{X}=\frac{1}{n}\sum_{i=1}^{n}[X_{i}-E(X)]{\left[X_{i}-E(X)\right]}^{T}$$

Then, the random weighting estimation of ${\hat{\sum }}_{X}$ is

(17) $${\hat{\sum }}_{X}^{*}=\sum_{i=1}^{n}v_{i}[X_{i}-E(X)]{\left[X_{i}-E(X)\right]}^{T}$$

## 4. Adaptive Kalman Filtering

### 4.1. Conventional Kalman Filter

As an efficient and recursive estimator, Kalman filter has been widely used for eliminating the random noise of FOG sensor [18,25,28,31]. It is a set of mathematical equations to estimate the state of system and minimize the mean squared error of residuals using the prior knowledge about dynamic process and measurement models, in addition to the process and measurement noise. Let the linear dynamic system and measurement equations be given by

(18) $$X_{k}=\Phi_{k,k-1}X_{k-1}+\Gamma_{k-1}W_{k-1}$$

(19) $$Z_{k}=H_{k}X_{k}+V_{k}$$

where *X_k_* is the state vector at epoch *k*, *Z_k_* is measurement vector, *Φ_k_,_k_*_-1_ is the state transition matrix, *Γ_k__-_*_1 _is the system noise driving matrix, and *H_k_* denotes the measurement matrix. *W_k_* is the state noise with covariance matrix $\sum_{W_{k}}$, *V_k_* is the measurement noise with covariance matrix $\sum_{V_{k}}$. *W_k_* and *V_k_* are assumed to be discrete white Gaussian noise with zero mean, known distributions and uncorrelated to each other, satisfying

(20) $$\{\begin{array}{c} \begin{array}{cc} E\left[W_{k}\right]=0 & E\left[W_{k}W_{j}^{T}\right]=Q_{k}\delta_{kj} \end{array}\\ \begin{array}{cc} E\left[V_{k}\right]=0 & E\left[V_{k}V_{j}^{T}\right]=R_{k}\delta_{kj} \end{array}\\ E\left[W_{k}V_{j}^{T}\right]=0 \end{array}$$

(21) $$\delta_{kj}=\{\begin{array}{c} 1\\ 0 \end{array}\begin{array}{cc} & \begin{array}{c} k=j\\ k\neq j \end{array} \end{array}$$

where *Q*_k_ is process noise covariance matrix, *R*_k_ is measurement noise variance matrix, and *δ*_kj_ is the Kronecker *δ* function. The CKF algorithm is described by the following equations

(22) $${\hat{X}}_{k,k-1}=\Phi_{k,k-1}{\hat{X}}_{k-1}$$

(23) $$P_{k,k-1}=\Phi_{k,k-1}P_{k-1}\Phi_{k,k-1}^{T}+\Gamma_{k-1}Q_{k-1}\Gamma_{k-1}^{T}$$

(24) $$K_{k}=P_{k,k-1}H_{k}^{T}{\left(H_{k}P_{k,k-1}H_{k}^{T}+R_{k}\right)}^{-1}$$

(25) $${\hat{X}}_{k}={\hat{X}}_{k,k-1}+K_{k}(Z_{k}-H_{k}{\hat{X}}_{k,k-1})$$

(26) $$P_{k}=(I-K_{k}H_{k})P_{k,k-1}$$

### 4.2. Random Weighting Estimation for Kalman Gain

The predicted state vector is

(27) $${\hat{X}}_{k,k-1}=\Phi_{k,k-1}{\hat{X}}_{k-1}$$

Accordingly, the innovation vector may be written as

(28) $$V_{{\hat{X}}_{k,k-1}}=Z_{k}-H_{k}{\hat{X}}_{k,k-1}$$

The variance of $V_{{\hat{X}}_{k,k-1}}$ is expressed as

(29) $${\hat{\sum }}_{V_{{\hat{X}}_{k,k-1}}}=\frac{1}{N}\sum_{i=1}^{N}[V_{{\hat{X}}_{k-i,k-i-1}}-E(V_{{\hat{X}}_{k-i,k-i-1}})]{\left[V_{{\hat{X}}_{k-i,k-i-1}}-E(V_{{\hat{X}}_{k-i,k-i-1}})\right]}^{T}$$

where $E(V_{{\hat{X}}_{k-i,k-i-1}})$ = 0. Accordingly, the random weighting estimation of ${\hat{\sum }}_{V_{{\hat{X}}_{k,k-1}}}$ is

(30) $${\hat{\sum }}_{V_{{\hat{X}}_{k,k-1}}}^{*}=\sum_{i=1}^{N}v_{i}[V_{{\hat{X}}_{k-i,k-i-1}}-E(V_{{\hat{X}}_{k-i,k-i-1}})]{\left[V_{{\hat{X}}_{k-i,k-i-1}}-E(V_{{\hat{X}}_{k-i,k-i-1}})\right]}^{T}=\sum_{i=1}^{N}v_{i}V_{{\hat{X}}_{k-i,k-i-1}}V_{{\hat{X}}_{k-i,k-i-1}}^{T}$$

The variance of $V_{{\hat{X}}_{k,k-1}}$ can be rewritten as

(31) $$\begin{array}{cc} \sum_{V_{{\hat{X}}_{k,k-1}}} & =E\left\{[V_{{\hat{X}}_{k,k-1}}-E(V_{{\hat{X}}_{k,k-1}})]{\left[V_{{\hat{X}}_{k,k-1}}-E(V_{{\hat{X}}_{k,k-1}})\right]}^{T}\right\}\\ & =E\left\{[Z_{k}-E(Z_{k})]{\left[Z_{k}-E(Z_{k})\right]}^{T}\right\}+E\left\{[E(H_{k}{\hat{X}}_{k,k-1})-H_{k}{\hat{X}}_{k,k-1}]{\left[E(H_{k}{\hat{X}}_{k,k-1})-H_{k}{\hat{X}}_{k,k-1}\right]}^{T}\right\}\\ & =\sum_{Z_{k}}+H_{k}(\sum_{{\hat{X}}_{k,k-1}})H_{k}^{T}=H_{k}P_{k,k-1}H_{k}^{T}+R_{k} \end{array}$$

By substituting Equation (31) into (24) and considering (30), the modified gain *K*_k_ can be described as

(32) $$K_{k}^{*}=P_{k,k-1}H_{k}^{T}{\left({\hat{\sum }}_{V_{{\hat{X}}_{k,k-1}}}^{*}\right)}^{-1}$$

### 4.3. Random Weighting Estimation for Covariance Matrix of Predicted State Vector

In KF prediction stage, the covariance matrix of predicted state vector is

(33) $$P_{k,k-1}=\Phi_{k,k-1}P_{k-1}\Phi_{k,k-1}^{T}+\Gamma_{k-1}Q_{k-1}\Gamma_{k-1}^{T}$$

That is

(34) $$\sum_{{\hat{X}}_{k,k-1}}=\Phi_{k,k-1}\sum_{{\hat{X}}_{k\text{-}1}}\Phi_{k,k-1}^{T}+\Gamma_{k-1}\sum_{W_{k-1}}\Gamma_{k-1}^{T}$$

By Equation (34), the covariance matrix of *W*_k-1_ can be written as

(35) $$\Gamma_{k-1}\sum_{W_{k-1}}\Gamma_{k-1}^{T}\text{=}\sum_{{\hat{X}}_{k,k\text{-}1}}-\Phi_{k,k\text{-}1}\sum_{{\hat{X}}_{k\text{-}1}}\Phi_{k,k\text{-}1}^{T}=\sum_{{\hat{X}}_{k,k\text{-}1}-{\hat{X}}_{k}}+\sum_{{\hat{X}}_{k}}-\Phi_{k,k\text{-}1}\sum_{{\hat{X}}_{k\text{-}1}}\Phi_{k,k\text{-}1}^{T}$$

Take the average of $\sum_{W_{k-i\text{-}1}}$ as the estimation of $\sum_{W_{k-1}}$ yields

(36) $$\Gamma_{k\text{-}1}{\hat{\sum }}_{W_{k-1}}\Gamma_{k-1}^{T}=\frac{1}{N}\sum_{i=1}^{N}\Gamma_{k\text{-}i\text{-}1}\sum_{W_{k-i-1}}\Gamma_{k\text{-}i\text{-}1}^{T}$$

Considering Equation (35), the random weighting estimation of $\sum_{W_{k-1}}$ is

(37) $$\begin{array}{ll} \Gamma_{k\text{-}1}{\hat{\sum }}_{W_{k-1}}^{*}\Gamma_{k-1}^{T} & =\sum_{i=1}^{N}v_{i}\Gamma_{k\text{-}i\text{-}1}\sum_{W_{k-i-1}}\Gamma_{k\text{-}i\text{-}1}^{T}\\ & =\sum_{i=1}^{N}v_{i}(\sum_{{\hat{X}}_{k-i,k-i-1}-{\hat{X}}_{k-i}}+\sum_{{\hat{X}}_{k-i}}-\Phi_{k-i,k-i-1}\sum_{{\hat{X}}_{k-i-1}}\Phi_{k-i,k-i-1}^{T})\\ & ={\hat{\sum }}_{{\hat{X}}_{k,k\text{-}1}-{\hat{X}}_{k}}^{*}+{\hat{\sum }}_{{\hat{X}}_{k}}^{*}-\Phi_{k,k\text{-}1}{\hat{\sum }}_{{\hat{X}}_{k\text{-}1}}^{*}\Phi_{k,k\text{-}1}^{T} \end{array}$$

Accordingly, we have

(38) $$\Gamma_{k\text{-}1}{\hat{Q}}_{k-1}^{*}\Gamma_{k\text{-}1}^{T}={\hat{\sum }}_{{\hat{X}}_{k,k\text{-}1}-{\hat{X}}_{k}}^{*}+{\hat{\sum }}_{{\hat{X}}_{k}}^{*}-\Phi_{k,k\text{-}1}{\hat{\sum }}_{{\hat{X}}_{k\text{-}1}}^{*}\Phi_{k,k\text{-}1}^{T}$$

Take the average of $\sum_{{\hat{X}}_{k\text{-}i,k\text{-}i-1}}$ as the estimation of $\sum_{{\hat{X}}_{k,k-1}}$ yields

(39) $${\hat{\sum }}_{{\hat{X}}_{k,k-1}}\text{=}\frac{1}{N}\sum_{i=1}^{N}\sum_{{\hat{X}}_{k-i,k-i-1}}$$

The random weighting estimation of $\sum_{{\hat{X}}_{k,k-1}}$ is

(40) $${\hat{\sum }}_{{\hat{X}}_{k,k-1}}^{*}\text{=}\sum_{i=1}^{N}v_{i}\sum_{{\hat{X}}_{k-i,k-i-1}}$$

Substituting Equation (34) into (40), we have

(41) $${\hat{\sum }}_{{\hat{X}}_{k,k-1}}^{*}\text{=}\sum_{i=1}^{N}v_{i}(\Phi_{k\text{-}i,k\text{-}i\text{-}1}\sum_{{\hat{X}}_{k\text{-}i\text{-}1}}\Phi_{k\text{-}i,k\text{-}i\text{-}1}^{T}+\Gamma_{k\text{-}i\text{-}1}Q_{k\text{-}i\text{-}1}\Gamma_{k\text{-}i\text{-}1}^{T})=\Phi_{k,k\text{-}1}{\hat{\sum }}_{{\hat{X}}_{k\text{-}1}}^{*}\Phi_{k,k\text{-}1}^{T}+\Gamma_{k\text{-}1}{\hat{Q}}_{k-1}^{*}\Gamma_{k\text{-}1}^{T}$$

Substituting Equation (38) into (41), we have

(42) $${\hat{\sum }}_{{\hat{X}}_{k,k-1}}^{*}=\Phi_{k,k\text{-}1}{\hat{\sum }}_{{\hat{X}}_{k\text{-}1}}^{*}\Phi_{k,k\text{-}1}^{T}+{\hat{\sum }}_{{\hat{X}}_{k,k\text{-}1}-{\hat{X}}_{k}}^{*}+{\hat{\sum }}_{{\hat{X}}_{k}}^{*}-\Phi_{k,k\text{-}1}{\hat{\sum }}_{{\hat{X}}_{k\text{-}1}}^{*}\Phi_{k,k\text{-}1}^{T}={\hat{\sum }}_{{\hat{X}}_{k,k\text{-}1}-{\hat{X}}_{k}}^{*}+{\hat{\sum }}_{{\hat{X}}_{k}}^{*}$$

The error question of predicted state vector can be defined as

(43) $$V_{{\bar{X}}_{k}}\text{=}{\hat{X}}_{k,k-1}-{\hat{X}}_{k}$$

The random weighting estimation of $\sum_{V_{{\bar{X}}_{k}}}$is

(44) $${\hat{\sum }}_{V_{{\bar{X}}_{k}}}^{*}=\sum_{i=1}^{N}v_{i}[V_{{\bar{X}}_{k-i}}-E(V_{{\bar{X}}_{k-i}})]{\left[V_{{\bar{X}}_{k-i}}-E(V_{{\bar{X}}_{k-i}})\right]}^{T}$$

The random weighting estimation of $\sum_{{\hat{X}}_{k}}$ is

(45) $${\hat{\sum }}_{{\hat{X}}_{k}}^{*}\text{=}\sum_{i=1}^{N}v_{i}[{\hat{X}}_{k-i}-E({\hat{X}}_{k-i})]{\left[{\hat{X}}_{k-i}-E({\hat{X}}_{k-i})\right]}^{T}$$

Considering Equations (42)–(45), the covariance matrix of predicted state vector ${\hat{\sum }}_{{\hat{X}}_{k,k-1}}^{*}$ can be updated by RWE as follows

(46) $$\begin{array}{ll} {\hat{\sum }}_{{\hat{X}}_{k,k-1}}^{*} & ={\hat{\sum }}_{{\hat{X}}_{k,k\text{-}1}-{\hat{X}}_{k}}^{*}+{\hat{\sum }}_{{\hat{X}}_{k}}^{*}={\hat{\sum }}_{V_{{\bar{X}}_{k}}}^{*}+{\hat{\sum }}_{{\hat{X}}_{k}}^{*}\\ & =\sum_{i=1}^{N}v_{i}[V_{{\bar{X}}_{k-i}}-E(V_{{\bar{X}}_{k-i}})]{\left[V_{{\bar{X}}_{k-i}}-E(V_{{\bar{X}}_{k-i}})\right]}^{T}+\sum_{i=1}^{N}v_{i}[{\hat{X}}_{k-i}-E({\hat{X}}_{k-i})]{\left[{\hat{X}}_{k-i}-E({\hat{X}}_{k-i})\right]}^{T} \end{array}$$

**Figure 2:**
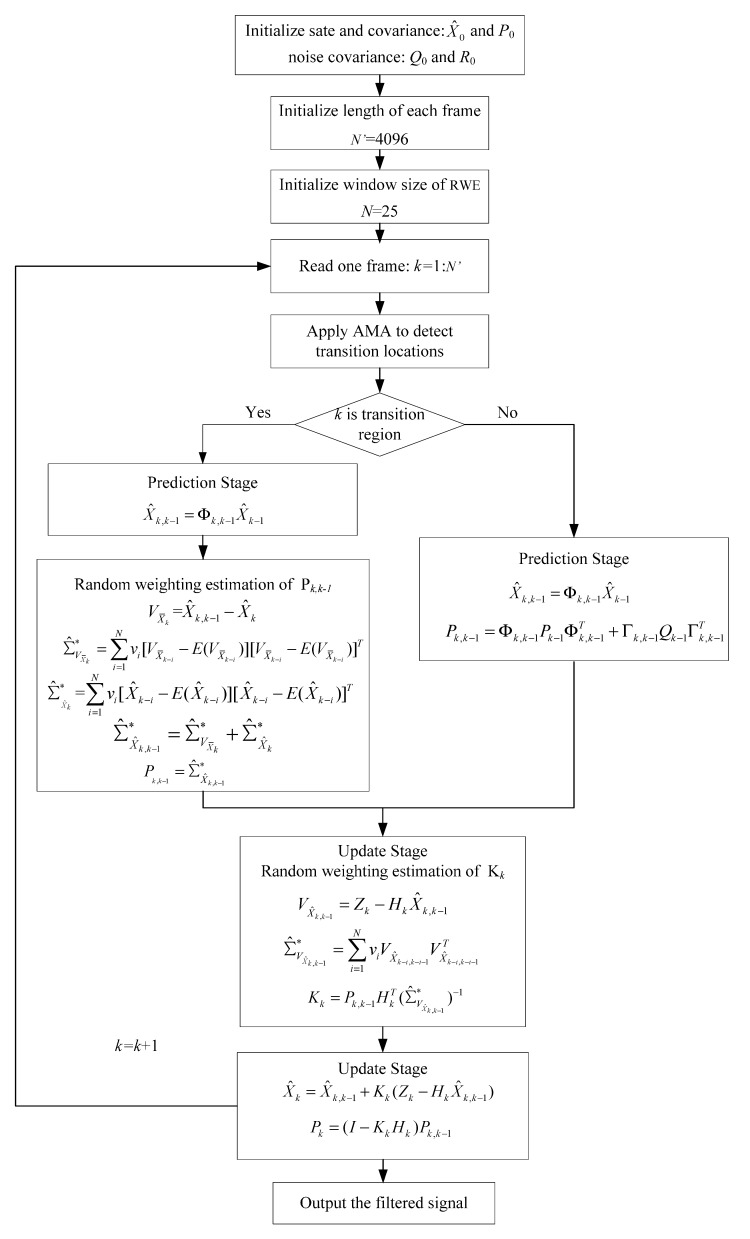
Operation of the proposed AMA-RWE-DFAKF algorithm.

KF parameters *Q*_k_, *R*_k_, and *P*_k_ impact not only on the noise level of output signal, but also on the inertia of KF response [27]. The inertia increases with the value of *R*_k_ while the noise level decreases. Conversely, the inertia decreases with the increase of value of *Q*_k_ while the noise level increases. In the dynamic case, these fixed values are critical in KF denoising scheme due to the noise characteristic of the FOG is more complex and time varying. To adjust KF parameters for the real-time applications, a double-factor adaptive KF combined AMA and RWE as shown in Figure 2, called AMA-RWE-DFAKF, is proposed for denoising the FOG signal. The Kalman gain is updated as Equation (32) in any region and the covariance matrix of predicted state vector is modified as Equation (46) only in transition region, which in fact has the same adaptive filter mechanism as [17], except that different methods are used to estimate these adaptive parameters.

## 5. Experimental Results and Discussions

**Figure 3:**
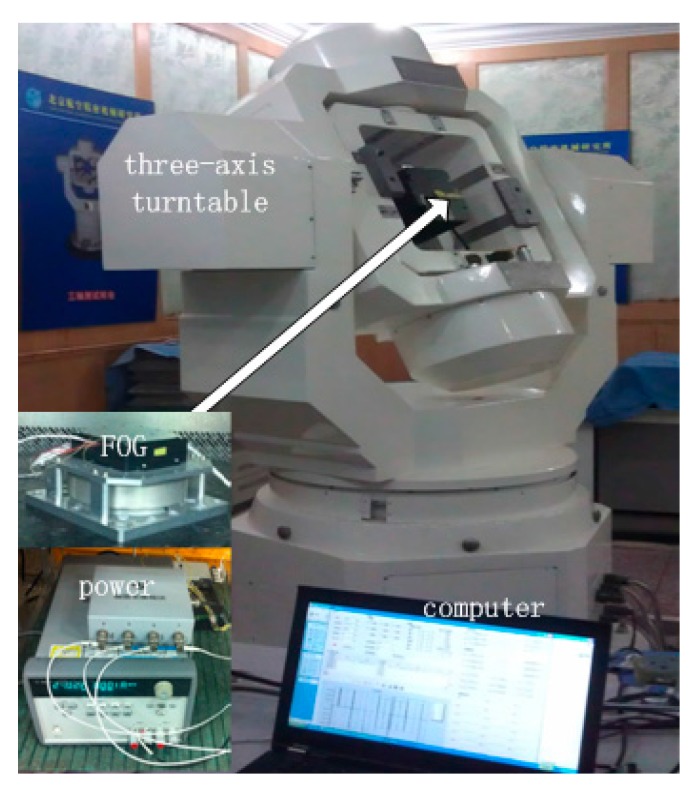
Experimental setup of FOG.

In this experiment, we test the filtering on real FOG signal under both static and dynamic environments. The experimental setup is shown in Figure 3. The setup has a single-axis FOG, three-axis turntable, power supply for FOG, and data processing computer. In the static condition, FOG is in zero rotation at room temperature, whereas in the dynamic condition, FOG is mounted on the three-axis turntable with different rotation rates.

### 5.1. Static Test Analysis

**Figure 4:**
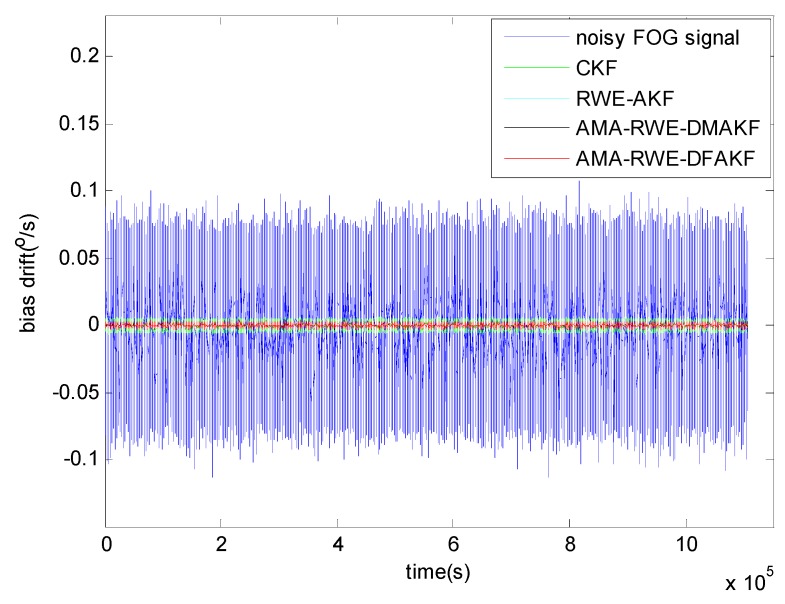
Denoising results of FOG signal in static condition.

Under room temperature, the FOG static data is recorded for 3 h with a sampling frequency of 100 Hz as shown in Figure 4. In KF, an AR (2) model is established as the system state equation using the first 6000 samples. Here, initial state and error covariance matrix of the state are assumed to ${\hat{X}}_{0}={\left[0,\;0\right]}^{T}$ and *P*_0_ = *diag*([1, 1]). H = [1, 0] is the observation matrix. And the measurement (*R*) and process (*Q*) noise covariance matrix are initialized to 0.01 and 0.0001, respectively. All these considered algorithms have the same parameters. Details on RWE-AKFG and AMA-DMKF are fully available in [23,24,25,26]. In this paper, *Q/R* is chosen as 0.01 (*k*1) and 0.1 (*k*2) for non-discontinuity region and discontinuity region, respectively. As discussed in [27], the value of *R* is fixed. AMA-RWE-DMAKF is that Kalman gain is updated by the covariance matrix of innovation sequence using random weighting method, which is a combination of RWE-AKFG and AMA-DMKF. For AMA-RWE-DFAKF and AMA-RWE-DMAKF algorithms, we considered 4096 samples as one frame. However, for RWE-AKFG and CKF we denoised the signal sample by sample. There is no discontinuity location detected by AMA because of collecting FOG signal in motionless environment.

**Figure 5:**
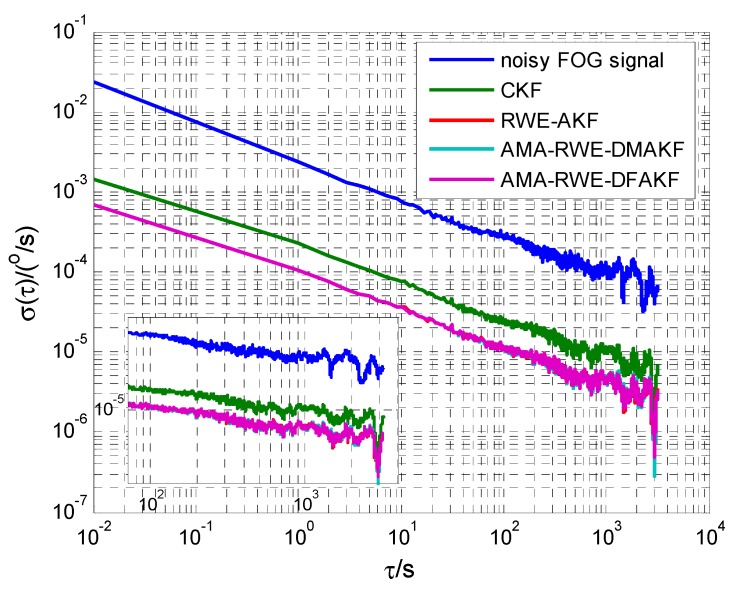
Allan variance analysis of FOG signal in static condition.

Allan variance is a popular technique to quantify and identify the random noise like quantization noise (Q), angle random walk (N), bias instability (B), rate random walk (K) and rate ramp (R) before and after denoising the FOG static drift signal [11,26]. In Allan variance curve, different slope corresponds to different random noise. To compare the denoising performance, Allan variance curves are plotted in Figure 5. It can be seen that the curves after filtering by these four methods decline in some degree and the curve from the proposed method is the lowest. In Figure 5, slopes of −1/2 and 0 indicate the present of angle random walk and bias instability in this FOG signal. The random error values are tabulated in Table 1. It is seen that the angle random walk and bias instability are reduced by 100 times as compared to the original value. Moreover, AMA-RWE-DFAKF, AMA-RWE-DMAKF, and RWE-AKFG algorithms give a competitive performance in denoising FOG static signal, and these results have a clear advantage as compared with CKF.

**Table 1:** Allan variance analysis results of FOG signal in static condition.

Methods	*Q* (*μrad*)	*N* (*o*/$\sqrt{h}$)	*B* (*o*/*h*)	*K* (*o*/h32)	*R* (*o*/*h*^2^)
Input	-	2.829 × 10^−3^	4.065 × 10^−4^	-	-
CKF	-	1.446 × 10^−4^	4.969 × 10^−6^	-	-
RWE-AKFG	-	6.702 × 10^−5^	2.398 × 10^−6^	-	-
AMA-RWE-DMAKF	-	6.711 × 10^−5^	2.282 × 10^−6^	-	-
AMA-RWE-DFAKF	-	6.705 × 10^−5^	2.264 × 10^−6^	-	-

### 5.2. Dynamic Test Analysis

**Figure 6:**
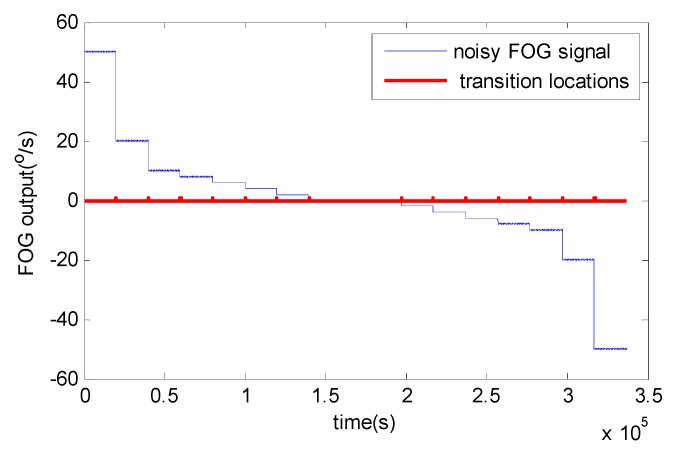
Detected discontinuities using AMA.

For acquiring the dynamic FOG data, the three-axis turntable is used to generate series of reference angular rate in the dynamic ranges, *i.e.*, ±10, ±20, and ±50°/s. In this study, the signal data is recorded for 1 h at room temperature with sampling frequency of 100 Hz shown in Figure 6 through a clockwise and counter-clockwise turntable rotation. The rotation rate is decreased in a stepwise manner starting from +50°/s and varying between 0°/s and 50°/s. An AR (2) model is established as the system state equation using the first 6000 differentiated samples. Here, the initial state and error covariance matrix of the state are assumed to ${\hat{X}}_{0}$ = [0, 0, 0]^T^ and *P*_0_ = diag([1, 1, 1]), respectively. The observation matrix becomes H = [1, 0, 0]. The initial values of *R* and *Q* are fixed to 0.01 and 0.0001 as mentioned above. The ratio between *Q* and *R* is switched from two states 0.01 and 0.1 for AMA-RWE-DMAKF.

As already mentioned, the signal is divided into frames with *N’* = 4096 samples. For each frame, AMA is used to detect discontinuity locations shown in Figure 6. From these results, the effectiveness of AMA is proved. These four algorithms are applied to denoise FOG dynamic signal with the same initial parameters chosen as in the static condition, *i.e.*, the measurement and process noise covariance matrix. Although RWE-AKFG and AMA-RWE-DMAKF give quite competitive results with AMA-RWE-DFAKF in static condition, but these fail to denoise signal in dynamic condition.

**Figure 7:**
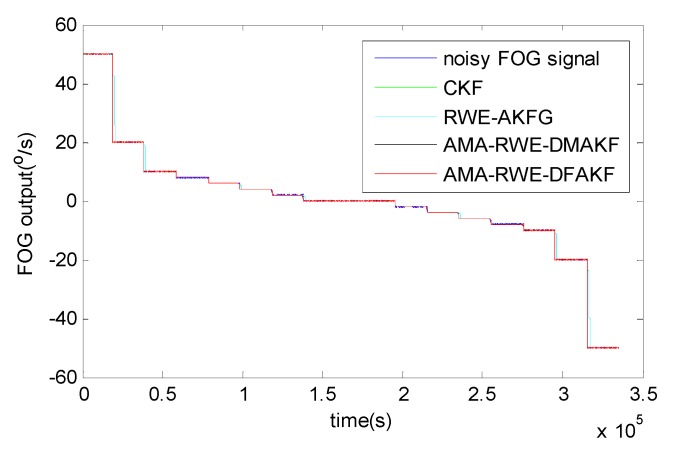
Denoising results of FOG dynamic signal.

**Figure 8:**
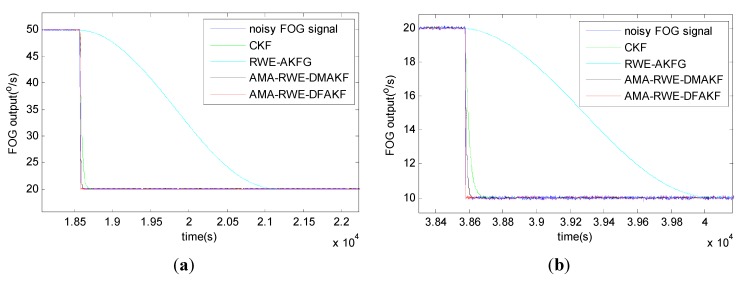

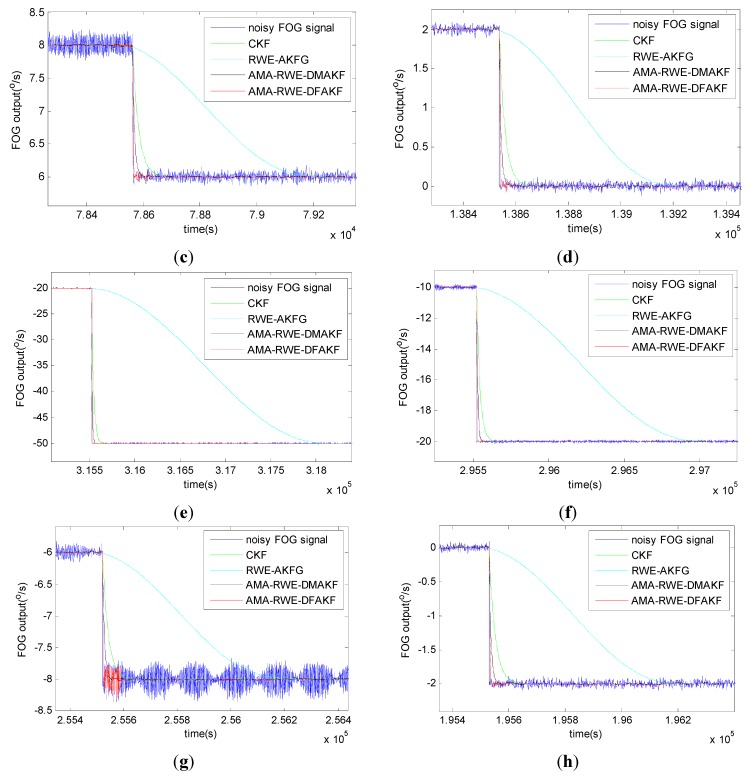
Comparison of denoising results for FOG dynamic signal at different rotations. (**a**) Rotation rate from 50 to 20°/s; (**b**) Rotation rate from 20 to 10°/s; (**c**) Rotation rate from 8 to 6°/s; (**d**) Rotation rate from 2 to 0°/s; (**e**) Rotation rate from −20 to −50°/s; (**f**) Rotation rate from −10 to −20°/s; (**g**) Rotation rate from −6 to −8°/s; (**h**) Rotation rate from 0 to −2°/s.

The denoising results for all samples are shown in Figure 7. To obtain a clear visuality, we have plotted only a portion of the signal in Figure 8 where we can compare the inertia of these four algorithms in the different transition. From Figure 8, we can see there exists some delay for every filtering in following trend of the signal. It is not acceptable for RWE-AKF due to the increase of the value of *R* while *Q* is fixed. Thus AMA-RWE-DMAKF with two Kalman gain *k*1 and *k*2 is developed to decrease the inertial, but it is difficult to pre-design these parameters. However, the proposed AMA-RWE-DFAKF algorithm can satisfy the lowest noise level and the lowest inertial by only adjusting the two parameters adaptively as described in Figure 2. Figure 7 and Figure 8 indicate that the AMA-RWE-DFAKF denosies the FOG dynamic signal better than all other algorithms.

**Figure 9:**
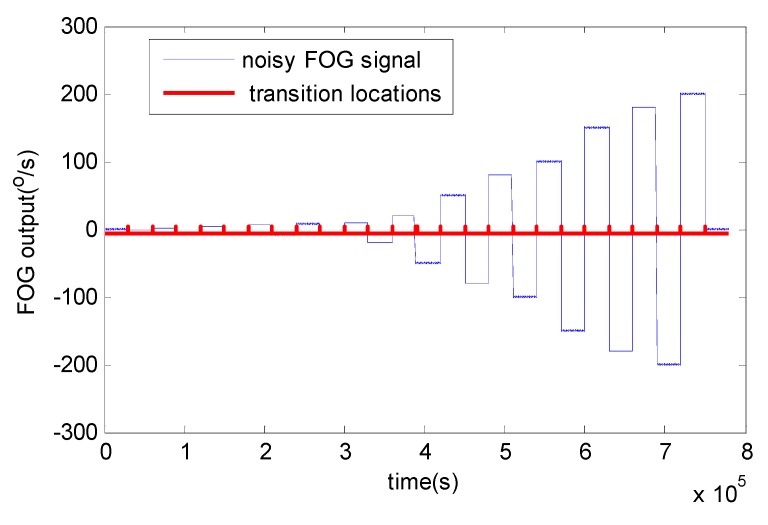
Detected discontinuities using AMA.

**Figure 10:**
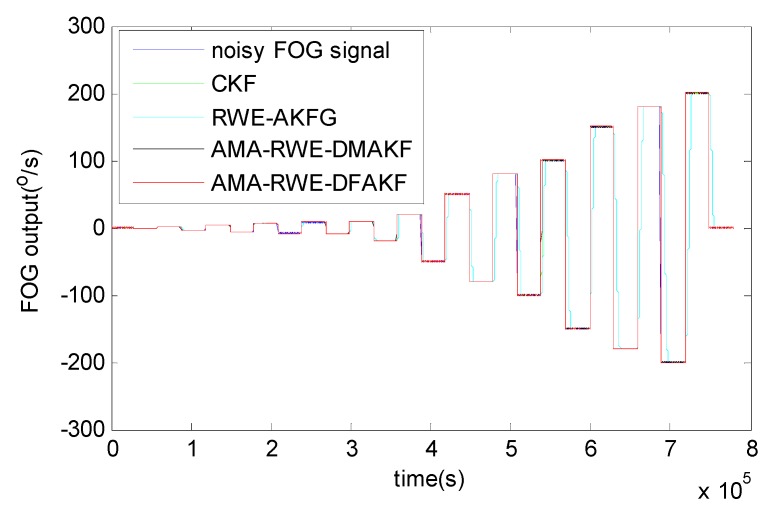
Denoising results of FOG dynamic signal.

**Figure 11:**
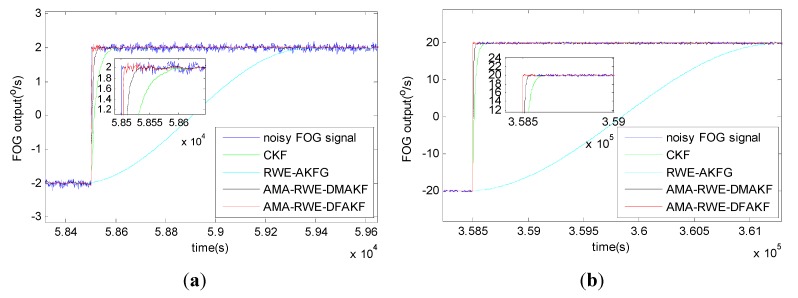

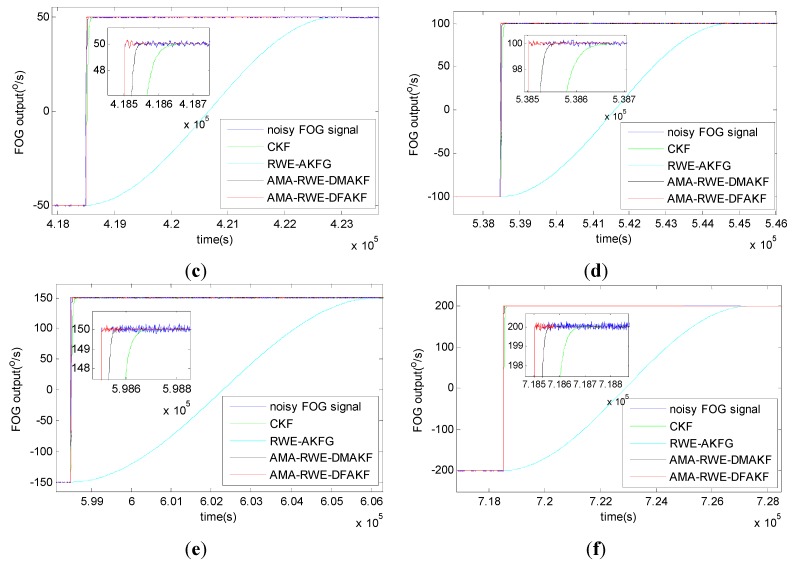
Comparison of denoising results for FOG dynamic signal at different rotations. (**a**) Rotation rate from −2 to 2°/s; (**b**) Rotation rate from −20 to 20°/s; (**c**) Rotation rate from −50 to 50°/s; (**d**) Rotation rate from −100 to 100°/s; (**e**) Rotation rate from −150 to 150°/s; (**f**) Rotation rate from −200 to 200°/s.

To further verify the effectiveness of AMA-RWE-DFAKF in denoising the FOG dynamic signal, we apply the same procedures on another FOG made in different company. The rotation rate of the table is increased by the alternatively positive and negative variations from 0°/s to 200°/s. Step signals data are collected for 1 h with sampling frequency of 200 Hz shown in Figure 9 and Figure 10. For comparing denoising results clearly, the zoomed figures of denoised signal in different rotation rates are plotted in Figure 11. The novel AMA-RWE-DFAKF has the minimum lag at transition times, which can satisfy both conditions of the lowest noise level and the lowest inertia.

Mean square error (MSE), root mean square error (RMSE), or signal-to-noise power ratio (SNR) [11,31] are generally employed to compare the performance of different denoising methods before and after denoising the FOG dynamic drift signal. The RMSE is defined as follows

(47) $$RMSE=\sqrt{\frac{1}{N}\sum_{t=1}^{N}{\left(x(t)-\hat{x}(t)\right)}^{2}}$$

where $\hat{x}(t)$ is the denoised signal, *x*(*t*) is the actual signal and *N* is the number of signal.

The RMSE results are calculated before and after denoising in Table 2 and Table 3. It is observed that the AMA-RWE-DFAKF has the minimum RMSE compared with other algorithms. Thus the effectiveness of this improved algorithm is verified in denoising FOG signal under both static and dynamic conditions.

**Table 2:** RMSE results of ±50°/s FOG signal with sampling 100 HZ.

Rotation (°/s)	Input	CKF	RWE-AKFG	AMA-RWE-DMAKF	AMA-RWE-DFAKF
+50	0.0384	0.0055	0.0040	0.0040	0.0040
+20	0.0462	0.0071	4.6724	0.0056	0.0038
+10	0.0573	0.0067	0.7951	0.0055	0.0036
+8	0.1145	0.0064	0.0103	0.0064	0.0032
+6	0.0515	0.0069	0.0071	0.0056	0.0037
+4	0.0403	0.0071	0.0095	0.0054	0.0038
+2	0.0358	0.0070	0.0097	0.0052	0.0037
0	0.0351	0.0073	0.0087	0.0054	0.0040
0	0.0349	0.0072	0.0039	0.0053	0.0039
−2	0.0366	0.0073	0.0102	0.0055	0.0040
−4	0.0441	0.0071	0.0102	0.0055	0.0046
−6	0.0657	0.0069	0.0102	0.0058	0.0037
−8	0.1552	0.0069	0.0117	0.0075	0.0040
−10	0.0778	0.0068	0.0071	0.0060	0.0037
−20	0.0402	0.0072	0.7679	0.0055	0.0039
−50	0.0369	0.0072	4.6649	0.0054	0.0039

**Table 3:** RMSE results of ±200°/s FOG dynamic signal with sampling 200 HZ.

Rotation(°/s)	Input	CKF	RWE-AKFG	AMA-RWE-DMAKF	AMA-RWE-DFAKF
0	0.0566	0.0087	0.0056	0.0056	0.0055
−2	0.0633	0.0165	0.0138	0.0082	0.0063
+2	0.0637	0.0163	0.0137	0.0081	0.0062
−4	0.1208	0.0233	0.0221	0.0087	0.0067
+4	0.1215	0.0234	0.0237	0.0150	0.0075
−6	0.1375	0.0205	0.0809	0.0186	0.0075
+6	0.1393	0.0208	0.1616	0.0187	0.0083
−8	0.1619	0.0210	0.2794	0.0176	0.0081
+8	0.1635	0.0204	0.4068	0.0170	0.0072
−10	0.1694	0.0215	0.5742	0.0175	0.0079
+10	0.1681	0.0197	0.7436	0.0163	0.0074
−20	0.1317	0.0239	1.8871	0.0213	0.0085
+20	0.1300	0.0226	3.3568	0.0201	0.0081
−50	0.1327	0.0174	8.8623	0.0156	0.0067
+50	0.1214	0.0161	15.5376	0.0153	0.0066
−80	0.1138	0.0118	23.1207	0.0107	0.0059
+80	0.1082	0.0117	31.3297	0.0109	0.0059
−100	0.1000	0.0114	37.0857	0.0098	0.0054
+100	0.0958	0.0111	43.3255	0.0096	0.0051
−150	0.0972	0.0100	59.1451	0.0096	0.0053
+150	0.0968	0.0102	76.2005	0.0097	0.0052
−180	0.1041	0.0098	86.9188	0.0086	0.0052
+180	0.1049	0.0099	97.8416	0.0087	0.0054
−200	0.1107	0.0096	105.2709	0.0085	0.0049
+200	0.1106	0.0109	112.7862	0.0088	0.0053
0	0.0563	0.0103	43.2174	0.0087	0.0055

**Figure 12:**
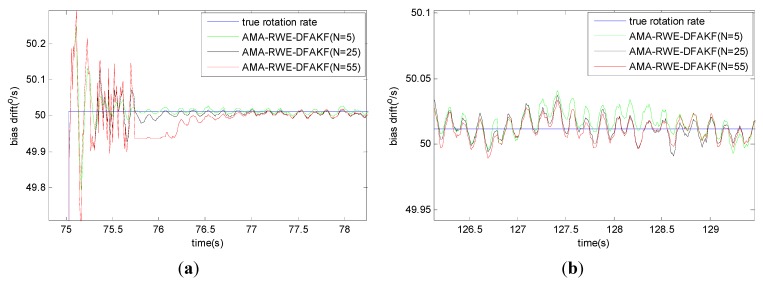
Comparison of denoising results for FOG signal with different parameters. (**a**) Comparison of convergence speeds; (**b**) Comparison of filtering accuracy.

**Figure 13:**
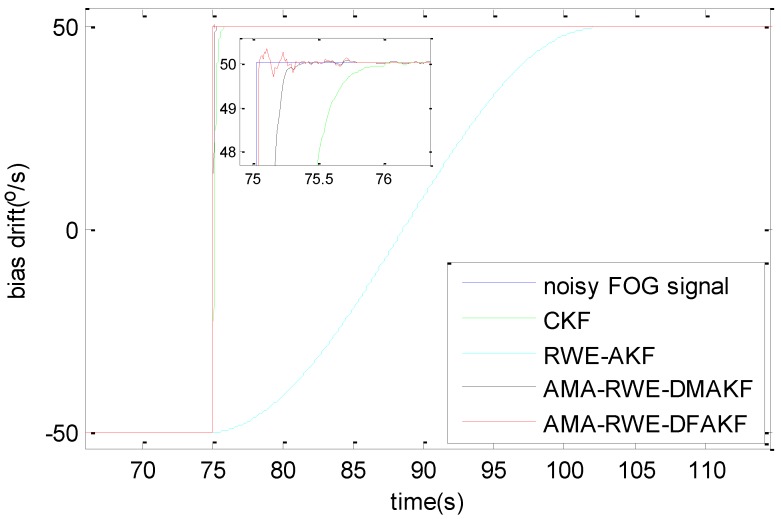
Comparison of denoising results for FOG dynamic signal.

Take the rotation rate from −50°/s to +50°/s as an example, the window size of RWE in AMA-RWE-DFAKF is discussed shown in Figure 12, because it can affect the convergence speed and filtering accuracy. The window of size is set to 5, 25, and 55, denoting small, middle, and long window. It can be seen that the smaller window size (N = 5) gives the faster convergence speed shown in Figure 12a, but has the poor filtering accuracy shown in Figure 12b. While, the longer window size (N = 55) give the higher filtering accuracy, but has the poor convergence speed. To get a relatively higher accuracy and faster convergence speed, the window size (N = 25) is chosen. The filter’s convergence speed shown in Figure 13 is about 0.2 s for AMA-RWE-DFAKF, 0.4 s for AMA-RWE-DMAKF, 1.2 s for CKF, and 30 s for RWE-AKF. Moreover, the proposed filter’s convergence speed is also faster compared with that of CKF, RWE-AKF, and AMA-RWE-DMAKF when the rotation rate is varying in other different values such as ±100°/s, ±150°/s and ±200°/s.

## 6. Conclusions

In this paper, an AMA-RWE-DFAKF algorithm is proposed to denoise FOG static and dynamic signals. AMA is used to detect the discontinuities and RWE is used to estimate the double-factor in KF. The first adaptive parameter is Kalman gain updated by using RWE of the covariance matrix of innovation sequence. The second adaptive parameter is the covariance matrix of predicted state vector introduced to decrease the inertia at the discontinuities. In static condition, the performance of AMA-RWE-DFAKF is competitive with RWE-AKFG and AMA-RWE-DMAKF, but superior to CKF. Based on Allan variance analysis, the random errors like angle random walk and bias instability are reduced by 100 times. In dynamic condition, the minimum RMSE obtained by AMA-RWE-DFAKF show that it performs better than all considered algorithms. The effectiveness of this method is validated in denoising the single-axis FOG signal in both static and dynamic conditions. The future work is to verify this filtering in three-axis FOG and implement it in hardware.
